# Physical and Sociocultural Community-Level Influences on Cigar Smoking among Black Young Adults: An In-Depth Interview Investigation

**DOI:** 10.3390/ijerph19084430

**Published:** 2022-04-07

**Authors:** Aaron Broun, Lilianna Phan, Danielle A. Duarte, Aniruddh Ajith, Bambi Jewett, Erin L. Mead-Morse, Kelvin Choi, Julia Chen-Sankey

**Affiliations:** 1 Milken Institute School of Public Health, George Washington University, Washington, DC 20052, USA; abroun@gwu.edu (A.B.); dduarte41@gwu.edu (D.A.D.); 2 Division of Intramural Research, National Institute on Minority Health and Health Disparities, Bethesda, MD 20814, USA; lilianna.phan@nih.gov (L.P.); bambi.jewett@nih.gov (B.J.); kelvin.choi@nih.gov (K.C.); 3 School of Medicine, University of Pittsburgh, Pittsburgh, PA 15201, USA; ajith.aniruddh@medstudent.pitt.edu; 4 School of Medicine, University of Connecticut, Farmington, CT 06032, USA; mead@uchc.edu; 5 Center for Tobacco Studies, Rutgers Biomedical and Health Sciences, New Brunswick, NJ 08901, USA

**Keywords:** cigar smoking, African American, community-level risk factors, health disparities, in-depth interviews, qualitative research

## Abstract

Black young adults experience disparately high rates of cigar use and its health consequences. Little research has explored community-level influences on cigar smoking in this population, especially concerning product-specific influences and cigar smokers’ perceptions. We conducted in-depth interviews with 40 Black young adult (ages 21–29) cigar smokers in the Washington, D.C. area and analyzed themes regarding physical and sociocultural community-level factors perceived to influence cigar use. Themes were further analyzed based on participants’ predominant cigar products (cigarillos, large cigars, blunts). Participants reported easy access to affordable cigarillos, widespread cigarillo sales and targeted marketing, norms of cigar and blunt smoking for stress relief, socialization, and cultural participation, and ubiquitous cigar and blunt smoking cues, all of which promoted cigar use in their communities. Future research should further explore how community-level influences contribute to disproportionate cigar use among Black young adults. Our findings suggest that programs and policies addressing physical and sociocultural community-level pro-smoking influences may help mitigate cigar smoking disparities.

## 1. Introduction

Use of cigar products (including large cigars, cigarillos, and filtered cigars) remains a significant public health and health disparities issue in the U.S. Unlike cigarette consumption, which has substantially declined in past decades, annual consumption of cigar products doubled from 2000 to 2012 (6−12 billion sticks) [1]. Racial/ethnic minority groups, especially Black/African American populations, are also more likely to smoke cigar products than White populations [2,3]. For example, in 2015–2016, Black adults were about 2.3 times, 2.7 times, and 2.0 times more likely than White adults to have smoked cigar products in the past 30 days, to smoke cigar products every day, and to smoke cigar products “fairly regularly,” respectively [2].

Additionally, Black young adults (ages 18–30) in the U.S. have the highest little cigar/cigarillo smoking prevalence (2.7%) of any age-racial/ethnic group [3]. The prevalence of all cigar product smoking was 1.5–3 times higher among Black young adults compared to those of other races, with greater disparities of 3–5 times in little cigar/cigarillo smoking prevalence [3]. Compared to White cigar product smokers, Black smokers also have about 2.3-times greater odds of smoking blunts [2], defined as cigar products in which some or all of the tobacco has been hollowed out and replaced with cannabis, within the past 12 months.

The disproportionately high use of cigar products among Black populations, especially Black young adults, may contribute to health disparities experienced by these populations. Research has shown that cigar product smoking may carry many of the same health risks as cigarette smoking [4], and that large cigars and cigarillos can contain higher concentrations of nicotine and carcinogens than cigarettes [5,6]. These studies also suggest that cigar product smoking, even among those who have never smoked cigarettes or those reporting no inhalation, is associated with negative health consequences, including lung cancer, oral cancer, other morbidities, and mortality [5,6,7]. Additionally, blunt smoking, an addictive substance use behavior, is associated with increased cannabis use [8], dependence on both tobacco and cannabis products [9], and risks of carbon monoxide exposure compared to tobacco-free cannabis use [10].

Given the health risks from cigar and blunt smoking and its potential impact on tobacco-related health disparities, understanding the risk factors that influence cigar product smoking among Black young adults is critical. This information can be used to inform public health programs and policies aimed at reducing cigar product smoking and related health disparities in this population. Previous research on factors associated with cigar product smoking among Black populations has several limitations. First, most of these studies investigated individual (e.g., knowledge and harm perceptions) and interpersonal level (e.g., family/peer use) risk factors rather than community-level influences [11,12,13,14]. Socio-ecological models provide a framework for investigating multiple levels of influence, including community-level influences—shared physical and sociocultural factors that can shape health and behavior among a group of people [15]. Applying a health equity lens to socio-ecological models, the National Institute on Minority Health and Health Disparities (NIMHD) Research Framework posits that racial and ethnic health disparities related to a risky health behavior may be explained at various levels of influence (individual, interpersonal, community, and societal) across a wide domain of factors (biological, behavioral, physical environment, sociocultural environment, and healthcare system) [16]. Physical environment influences may involve personal, household, school/work, and community environments and resources; sociocultural environment influences may involve family/peer and community norms and interpersonal and local structural discrimination [16]. Here, we expand on existing research by focusing on the impact of physical and sociocultural environmental factors at the community level.

Second, the studies that focused on community-level risk factors mainly examined the differing presence and density of cigar retailers in the neighborhood and the advertising appearing in these retailers across various racial/ethnic and/or age groups rather than focusing on Black young adults specifically [17,18,19,20]. Our qualitative study includes other types of community-level influences within a narrower population and adds the opportunity to explore how Black young adult cigar product smokers may perceive the connections between various community-level factors and their cigar product smoking behavior. Finally, these previous studies often examined one type of cigar product (e.g., cigarillos) or grouped all cigar products together, and did not separate blunt smoking from cigar smoking behavior. We expand on this work using product-specific interview prompts and distinct blunt use questions to specify particular influences of concern and identify potential differences by product type.

Therefore, in order to understand the various potential community-level influences on cigar product smoking behavior among Black young adults, we explored the physical and sociocultural contexts of Black young adults’ cigar product smoking behaviors in their communities. Specifically, through in-depth interviews with Black young adult cigar product users, we aimed to characterize social and environmental influences on different cigar products and blunt smoking with a focus on community-level influences.

## 2. Materials and Methods

### 2.1. Participants and Study Procedures

We conducted in-depth phone interviews with 40 non-Hispanic Black young adult cigar product smokers from May to June 2020. Participants were recruited by the research team through social media sites (Instagram, Facebook, and Craigslist), predominantly targeting participants residing in the Washington D.C. metropolitan area. Individuals were eligible to participate if they met the following criteria: (1) self-identified as non-Hispanic Black or African American; (2) 21 to 29 years old; (3) a current cigar product smoker (defined as smoking large cigars, cigarillos, or filtered cigars ≥4 times in the last 2 weeks) [14,21]; and (4) able to read and speak English. Research team members screened interested individuals over the phone. Current cigar product smoking status was confirmed during the screening by asking potential participants to describe the characteristics and brands of the products they currently smoked. The research staff who screened the participants were trained to recognize cigar product brands and types. Eligible participants then received an email containing a link to an online survey that included a consent form and a brief questionnaire. Participants provided informed consent prior to completing the questionnaire and answered questions on socio-demographic characteristics and tobacco use behavior. Participants were scheduled for phone interviews following their survey completion. Those who completed both the survey and phone interview received a $100 Amazon gift card. The study was approved by the National Institutes of Health Institutional Review Board (project #P204893).

### 2.2. Interview Instrument and Structure

A senior researcher trained in qualitative data collection conducted all in-depth interviews. The in-depth interview is an established qualitative research tool used to explore participant perspectives and their contexts [22]. Participants were first asked to describe the brands and characteristics (including cigar product type and flavors) of the cigar products and cigar smoking patterns (e.g., frequencies) they currently smoked. Participants were also asked to describe their current blunt smoking behavior after the interviewer defined blunt products to them and assured them that the information would be kept confidential. They were then asked by the interviewer to describe the context and environment relevant to cigar product smoking (purchasing and accessing cigar products in the community; exposure to cigar product marketing; cigar product smoking triggers; perceived community influences on cigar product smoking). Interviews also included questions related to quitting cigar product smoking and the perceived influence of cigar product policies. Participants who indicated that they smoked blunts were also asked about their blunt smoking behavior and its contexts. Additionally, participants mentioning cannabis use as part of their smoking culture or environmental context were probed to describe this element further. The interviews lasted between 45 and 60 min. Notes were taken by the moderator during the interviews, and data saturation was reached after interview #30 for overall content. Ten more interviews were conducted to gather additional data for allowing thematic analysis by various types of cigar products.

### 2.3. Data Analysis and Reporting

Phone interviews were audio-recorded upon participant consent and later transcribed verbatim. De-identified transcripts were analyzed on Dedoose (SocioCultural Research Consultants, LLC, Manhattan Beach, CA, USA), a web-based qualitative data management application. The coders used thematic analysis to organize and analyze the interview data [23,24]. Four study team members initially developed the codebook based on the interview guide and identified themes and subthemes from reviewing the data relevant to the overarching research questions.

Three coders underwent training to familiarize themselves with the codebook, then applied the codes to all transcripts independently. All interview codes related to cigar product use, access, and perceptions were further classified by product type (large cigars, cigarillos, filtered cigars, and blunts). Each transcript was independently coded by two coders who later reconciled any coding disagreements in discussion with the research team. The percentage agreement of the codes used for this analysis was moderate to high (81–95%), demonstrating satisfactory inter-coder reliability [25]. Finally, coded content was categorized, counted, and analyzed for themes and subthemes arising from the data by two researchers independently. Table 1 describes the codes and their definitions included in the codebook for thematic analysis.

In accordance with prior thematic analyses, the estimated frequency of participants reporting themes and subthemes was recorded and reported as “all” (100%), “most” (70–99%), “more than half” (51–69%), “some” (20–50%), and “a few” (1–19%) [26,27]. Representative quotes, along with the age and sex of the quoted participant, were reported for each theme. Due to the limited amount of interview data specific to filtered cigar smoking, themes specific to filtered cigars were not generated or reported. In addition, participants’ socio-demographic characteristics and tobacco use histories were quantitatively analyzed using Stata 16.0 (StataCorp LLC, College Station, TX, USA).

## 3. Results

### 3.1. Participant Characteristics

The average age of participants in the sample was 26 years old, and slightly more than half of the participants were female (*n* = 23) (Table 2). Past 30-day smoking of cigarillos, large cigars, blunts, and filtered cigars was reported by 36 (90.0%), 24 (60.0%), 23 (57.5%), and 7 (17.5%) participants, respectively. Eighteen (45.0%) participants reported blunts as their most frequently smoked cigar product in the past 30 days, followed by cigarillos (*n* = 16, 40.0%), large cigars (*n* = 4, 10.0%), and filtered cigars (*n* = 2, 5.0%). Additionally, past 30-day use of cigarettes, e-cigarettes, and hookah was reported by 23 (57.5%), 26 (65.0%), and 27 (67.5%) of participants, respectively.

### 3.2. Sales of Cigar Products in the Community

#### 3.2.1. Convenient Retailers with Abundant Cigarillo Selections

When asked where participants usually access their cigar products, most participants who regularly smoked cigarillos mentioned that they accessed cigarillos through retailers such as gas stations and corner stores in the communities they live in. More than half of the participants also explicitly mentioned that almost all retailers in the neighborhood sell cigarillos. These participants noted that gas stations and convenience stores where cigarillos are sold are abundant and easily accessible in their communities. When asked whether it was easy to find the cigar products that they currently smoked, most participants who regularly smoked cigarillos responded that it was extremely easy, and there was ample access to their preferred cigarillo brands and flavors.

*“It would be weird for a gas station to not have cigarillos or cigarettes.”*

[Female, 23 years]

*“You can buy them at any gas station, or anything like that.”*

[Male, 29 years]

*“I choose them [gas stations] because they’re reliable and they’re really the most convenient. I can count on being able to buy them for the most part when I go into a store.”*

[Female, 25 years]

*“Black & Milds, I purchase those everywhere; the gas station. Any gas station you could think of.”*

[Female, 26 years]

#### 3.2.2. Specialty Sources for Large Cigars

Some participants who frequently smoked large cigars mentioned that they purchased large cigars from gourmet cigar shops or cigar lounges located in their neighborhood. A few mentioned that sometimes they had to travel to nearby neighborhoods or towns to purchase large cigars. Some of the participants who frequently smoked both large cigars and cigarillos found that it was much easier to find cigarillo products they frequently smoked than large cigars.

*“[Large] cigars, I go to the regular cigar stores, the specialty cigar stores. They have them in even some convenience stores. With the cigarillos, I can pretty much get those anywhere. The gas station, any little small corner store or Wawa, 7-Eleven. Anything like that.”*

[Male, 29 years]

*“Sometimes I’ll just go and pop into a 7-Eleven or gas station for cigarillos, but for the [large] cigars I’ll go to David’s Cigars…If it’s a cigar lounge, they’ll roll them for you, cut it, put it in a humidor.”*

[Female, 28 years]

### 3.3. Marketing of Cigars at Community Locations, Social Media, and Events

#### 3.3.1. Cigarillo Advertising Concentrated in and around Retailers

When asked whether they had seen any cigar product-related marketing and promotions recently, most participants reported that they often saw cigarillo advertisements at local retailers and other community fixtures such as windows, storefronts, and light poles. Product posters and displays located at retailers, visible both outside and inside of the store, were the most commonly described types of advertisements.

*“In the store, gas stations, outside of the gas stations on the window, and on the corner store window. They promote them everywhere. On the light poles. Wherever they know people will see them, they’re visible to the public.”*

[Male, 28 years]

*“They’re everywhere. It may be as simple as an ad right there on the front of the door before you enter a Shell gas station, or maybe an ad on the window at 7-Eleven.”*

[Female, 26 years]

*“Yes, there are a lot of billboards. There are a lot of billboards around the neighborhood and stickers and pictures and sale pictures of the wraps and stuff, like two for $1.99 or three for $4.99.”*

[Male, 28 years]

*“It was placed more in front of the store versus in the back part where the rest of the other products usually are. It was more towards the front, so they were actually showing that these are new brands and flavors that came out if you wanted to try it.”*

[Female, 25 years]

#### 3.3.2. Advertising Appeal through Flavors and Discounts

When further asked what specific content or messages they saw, more than half of the participants who recalled cigar product advertisements reported that the materials that caught their attention were often promoting new brands and flavors and/or those that featured price promotions such as coupons, discounts, and savings. When asked what they thought about those advertising messages, some reported that they would like to be informed of the new products and flavors entering the market as well as the discounts. A few explicitly mentioned the advertisements that introduced new flavors prompted them to purchase and try the flavors.

*“I like the way they show the different types of flavors that they have, the different types of brands that you can get. The displays say a lot as far as the brands and the flavors or new flavors that come out.”*

[Female, 25 years]

*“It might be a sign saying like they sell this particular brand or they have these flavors.”*

[Female, 25 years]

*“Actually, after the ad it made me feel as though I wanted it, it looked cool. I’d never tried that before, so it gave me a sense of wanting to try it.”*

[Male, 22 years]

#### 3.3.3. Social Media and Event-Based Advertising

A few participants mentioned that they followed cigarillo brands (e.g., Backwoods and Swisher accounts) and/or brand ambassadors and influencers on social media and participated in official brand email subscriptions for price discounts and notifications about new flavors. A few also reported they had attended entertainment activities (e.g., concerts and festivals) that were sponsored by cigarillo companies; during these events, they met brand ambassadors who introduced them to new products and flavors and handed out free products for trial. Additionally, one participant mentioned that she had encountered salespeople giving away free cigarillo products in her neighborhood.

*“I follow a lot of different brands on social media, so I’ll see ads on social media; or I will get newsletters in my emails; or they might have marketing events where they have a sales rep or different things like that.”*

[Female, 26 years]

*“It’s cigar videos, I’d say, because I follow certain companies that make cigars. Instagram videos, basically, about new ones that come out.”*

[Male, 22 years]

*“You can be on Instagram and an ad will pop-up about Black & Milds. If you’ve typed it in your phone before, if you’ve done anything. Like me I look at cigar videos, I look up different types of things so it may pop-up on my Google.”*

[Female, 26 years]

*“And then one time they had the girls, they call the Swisher Sweet Girls, that were doing a sampling, a promo sampling… That’s another way that you can try it, they’re very affordable.”*

[Female, 21 years]

### 3.4. Community Norm of Smoking Cigars and Blunts

#### 3.4.1. Normalized Cigarillo and Blunt Use

When asked to share their perceptions of cigar product smoking culture in their communities, most participants reported widespread community acceptance and norms of smoking cigarillos and blunts. More than half of participants reported that most of the people they knew living in the community smoked cigar products, blunts or other tobacco products. Some also mentioned that when they grew up, they were told by family and community members that cigarillos and blunts are not as harmful to someone’s health as cigarettes.

*“Black and Milds, cigarettes, blunts, everything. You name it, they’re smoking it around here.”*

[Female, 25 years]

*“The people here where I live right now, they’ll smoke blunts outside during the day. They don’t care. That message kind of makes you think, ‘Hey, this is normal.’ I kind of feel that way. Even though I really know it’s not normal, it seems that way and eventually, you believe it.”*

[Male, 28 years]

*“Everybody that I live with smokes. All of my good friends, they all smoke. We probably use the same brand of stuff. My family smokes. I don’t know, it’s just everybody around me smokes.”*

[Female, 23 years]

#### 3.4.2. Cigarillo and Blunt Use Norms Driven by Community and Personal Stress

Some participants discussed cigarillo and blunt smoking as a response to stress. Specifically, they reported that people from their communities were constantly stressed out, and those who smoked cigarillos and blunts were perceived to smoke to relieve stress. Many of these participants explicitly tied this stress to financial hardship and/or work.

*“I think that’s the biggest reason why most people in my community smoke it, is because it’s the safest way to escape. I think that’s what any drug is, an escape… I can only guess some people may need to escape from their situation because they live in poverty and they don’t want to think about it or they have so many people that they need to take care of and it stresses them.”*

[Female, 28 years]

*“Well- I feel as though it makes me feel like they are stressing too, so that’s why they smoke too.”*

[Female, 25 years]

*“Yes, stress. Stress makes me want to smoke. Everybody stresses. I stress almost every day, so that’s the main reason. … I don’t have a job. I don’t have any money and I have an eight-year-old with no income.”*

[Female, 27 years]

#### 3.4.3. Social Reasons for Cigar Use

Some described that it is common for cigarillo smokers in their community to discuss, collect, and try a variety of cigarillo products. Some participants mentioned that cigar lounges located in their communities served as community gathering places for people to relax and socialize; a few further discussed the experience of becoming close friends with other cigar smokers they met at the lounges and smoking large cigars as a way to bond with each other.

*“Being around my sister and being around this community that I recently moved to, and people smoke cigars, they taught me about cigars. They taught me about the different types of tobacco, taught me about the different ways that the cigars are rolled, and I started getting into it because it’s fascinating to me, for one… Basically, it was a community of people around me that got me into it.”*

[Female, 26 years]

*“I think the smoking cigars in the neighborhood, a lot of people have expressed interest. They got started with, ‘I heard jazz music coming from this place and want to go in and check it out, and this guy was doing something with his cigar so I wanted to try one and I want to learn more about it.’”*

[Female, 28 years]

#### 3.4.4. Cigarillos, Blunts, and Cannabis in Black Hip-Hop Culture

A few participants explicitly associated cigar product and blunt smoking with Black hip-hop culture or Black cannabis culture. Some of those connected the culture closely with a few cigarillo brands that are normatively smoked as blunts (e.g., Backwoods), identifying artist-product associations and the resulting behavioral norms as elements of cultural knowledge.

*“The closest I will say about seeing endorsement for it are through celebrities or stuff like that. Celebrities who are known to smoke like Wiz Khalifa or Snoop Dogg who have their own brands and they promote different brands.”*

[Female, 28 years]

*“If an artist has an endorsement with Swisher Sweets, he’ll have a post showing a painting of the different flavors.”*

[Female, 27 years]

*“Just, for example, basically just the hip-hop Black culture. I guess the smoking weed culture, the smoking weed constituents, that type of scenario. Wiz Khalifa, he raps. Rappers, they say, ‘We smoke Backwoods.’”*

[Male, 29 years]

*“Keep smoking the blunt. Nobody’s smoking white papers. Especially Black boys. That’s what Tupac said.”*

[Male, 28 years]

### 3.5. Ubiquitous Cigar and Blunt Use Cues in the Community

#### 3.5.1. Smoking Cues and Signs of Use

When discussing cigar product smoking in their neighborhood or community, most participants also reported ubiquitous cigarillo and blunt smoking and smoking cues in the neighborhood. Frequently reported signs of cigar product and blunt smoking included wrappers, tips, litters, and smells of burning tobacco and cannabis in the neighborhood. Some participants also reported seeing others rolling up blunts on the street and congregating outside of certain community places (e.g., public parks) to smoke cigar products and blunts.

*“There are wrappers and cigarillo wrappers laying around everywhere: on the street, on the sidewalk, on the grass, in people’s yards, on people’s cars, on the roof. You might see a pack of Grabba Leaf on the roof. They roll in the streets, in the neighborhood.”*

[Male, 28 years]

*“Weed has a smell. Sometimes when I walk out of my apartment my hallways, my floor will smell like weed. Even if I’m outside sometimes I can smell the smell of weed. People will literally walk down the street smoking.”*

[Female, 23 years]

*“Yes, or in the house, on the porch. There are tons of balconies and you can smell it. If I were to take a walk around my neighborhood right now, I would pass three houses where I could smell weed or even see people smoking on the porch.”*

[Female, 28 years]

*“If there’s a lot of people walking up and down the street and you see…Sometimes I can see them, some of them are my neighbors so they’re on the neighboring patios and they’ll sit outside on their patios and smoke.”*

[Female, 21 years]

#### 3.5.2. Pervasive Cues Promote Continued Smoking

A few participants indicated that these signs of smoking cues commonly found in the neighborhood made them crave smoking cigar products and/or experience difficulties in quitting those products. A few participants also reported smoking cigar products and blunts to conform to smoking norms or perceived peer pressure.

*“There’s just so much smoking going on. That’s what got me into smoking…People who don’t have a strong willpower could be subjected to smoking more than what they want.”*

[Male, 27]

*“I guess there’s a certain element of just wanting to share in the same activity as your friends as well that makes you want to do it. Or if someone else is smoking and you’re in the same space, it’s just easy to say, ‘Hey, can I take a puff or two?’”*

[Female, 25 years]

*“But I try to stay away from other smokers because like I said, I’m trying to cut down. Sometimes it’s hard for me too.”*

[Female, 25 years]

*“It’s a trigger because if I see somebody have one I’m like, ‘I want to have one.’… I’ll be like, ‘I need to have one too.’ It’s a trigger. I don’t know why.”*

[Female, 28 years]

*“Usually, when they’re in party mode they like to pull out their blunts and cigars. When I see them do it, then I’ll go ahead and join in because I don’t want to be ridiculed by them for not doing it.”*

[Male, 25 years]

## 4. Discussion

Themes from in-depth interviews with Black young adult cigar smokers indicated that this group faces community-level pro-cigar-smoking influences in their physical and sociocultural environments. Black young adult cigar smokers reported easy access to affordable products, especially cigarillos, in stores throughout their neighborhoods. They described frequent exposure to cigar product advertising not only on storefronts and point-of-sale displays but also on social media and at events, with many participants finding cigar product discounts and flavor promotions especially appealing. Cigar product and blunt use were highly accepted and normalized in Black young adults’ communities, often providing a means for socialization as well as stress relief among community members facing economic strain. For some Black young adults, cigar product and blunt smoking norms were also connected to hip-hop culture. The ubiquity of cigar product and blunt smoking in participants’ communities yielded a plethora of pro-smoking cues that could promote continued use and hinder attempts at avoidance and cessation.

Participants’ emphasis on the ubiquity and affordability of cigar products, especially cigarillos, at local retailers highlights a distinct community-level facilitator of use. These findings are consistent with prior quantitative spatial analyses, which demonstrate stark neighborhood-level disparities in little cigar/cigarillo availability. For example, one study in Washington, D.C., found that the odds of little cigar/cigarillo availability were more than ten times greater at stores in neighborhoods with a high proportion of Black residents [18]. Accordingly, policies targeting product availability—for example, restricting the sale of single cigarillos—may be of interest in reducing cigar product use in those communities [28].

Our results show that consistent reports of widespread cigar marketing may serve as an important community-level influence among Black young adult cigar smokers. Studies have shown that tobacco marketing exposure may contribute to tobacco use among youth and young adults [29,30,31]. Most participants recalled seeing cigar advertisements throughout their communities, exposing them constantly to a risk factor for cigar use on a community level in their daily lives. Prior studies also indicate that cigar marketing is disproportionately targeted to neighborhoods with larger Black populations, lower incomes, and more young adults [17,18,19,32]. Therefore, our results and the past research suggest a need for placing advertising and marketing restrictions in high-risk communities to reduce these pro-cigar-smoking influences that are disparately experienced by Black young adult communities. For example, policies prohibiting cigar advertisements at the point of sale, which have shown promise in other countries, including Australia and Canada [33,34], may reduce Black and poor young adults’ disparate exposure to a pro-cigar-smoking influence. Additionally, existing national and local policies [35] that prohibit billboard marketing and brand sponsorships by cigarette companies should be expanded to explicitly cover cigar marketing.

Notably, our study participants’ emphasis on flavor-related content as a frequent and appealing advertising element is of health equity concern, given evidence that the tobacco industry has used flavored cigarillos and little cigars and their marketing to target young and Black audiences [32,36,37]. Accordingly, a recent U.S. FDA proposal to restrict the sale of flavored cigar products is a welcome development that could reduce Black young adults’ disproportionate exposure to flavored cigar product sales and marketing [38]. As new marketing strategies like social media advertising and brand ambassadors take hold among the same groups targeted by physical marketing in their communities, policies restricting the advertising of cigar products are another tool to combat smoking cues and disparities at a community level.

Participants also mentioned pervasive cigar product placements surrounding popular rappers and hip-hop artists. Sponsorships of artists and events have been a consistent element of the tobacco industry’s decades-long efforts to target Black young adults, often with specific products [39,40]. Modern cigarillo manufacturers’ forays into the hip-hop industry, such as the Backwoods promotions, rap battle sponsorships by the Al Capone brand, and the ongoing Swisher Sweets Artist Project, are reminiscent of earlier menthol cigarette marketing campaigns that targeted young Black males in the jazz and hip-hop scenes [39,40,41]. Black young adults connected community norms of cigar product and blunt smoking to industry-manufactured associations of these products with rap and hip-hop artists, lending further support to calls for regulation of cigar companies’ music industry sponsorships on the grounds of targeting young, Black populations.

In describing the widespread normalization of cigar product and blunt smoking in their communities, many participants also connected the behavior to stress that they perceived to be shared among many community members. Notably, prior research indicates that observed stress among others can increase one’s own stress [42], an observation that aligns with community development theorists’ suggestion that community conditions impact well-being across concentric individual, social, and ecological levels [43]. The compounding of societal stressors is thought to occur at both the individual and community levels [44], which could contribute to disparate vulnerabilities to tobacco use in pursuit of stress relief. Economic hardship, mentioned by several participants in our study, may represent a stressor faced disproportionately by the communities of Black young adults given their higher rates of poverty and lower median incomes compared to other racial and age groups [45]. Prior research has suggested that subjective socioeconomic status, as measured by self-reported financial difficulties, may contribute to cigarette smoking among Black adults [46]; further investigation is warranted to determine whether this association holds for cigar product use among Black young adults. More research is also needed to explore the potential associations between other stressors and cigar product use and should focus on those stressors that are likely to impact disadvantaged and industry-targeted groups like Black young adults. For example, self-reported racial discrimination has been linked to higher odds of use for both tobacco and cannabis among Black adults [47,48]. More research is needed to determine the role of financial and racial stresses in the etiology and exacerbation of cigar product smoking, specifically among Black communities. Our findings also demonstrate a need for effective programs to promote healthier coping behaviors within communities that experience chronic, excessive stress.

Furthermore, Black young adults in our sample described a variety of social and environmental cues (e.g., seeing other people smoking and rolling up blunts, seeing cigar product wrappers and tips, and smelling burning tobacco and cannabis) that could trigger cigar product smoking and hinder cessation. Exposure to tobacco use in the social, physical, and symbolic environments can serve as significant sources of normative influence to promote tobacco use [49]. Previous research suggests that social and physical environments (e.g., on social occasions or in stressful situations) may trigger behaviors like flavored cigar use [21] and cigar-cigarette dual use [50]. As some participants noted, social and environmental cues that trigger cigar smoking can act as a promoter of continued use and a barrier to cessation. Consistently, a previous analysis found that barriers to cigar smoking cessation among Black young adults included widespread cigar-smoking cues, easy availability of cigar products, and persistent stressors [51]. Since Black individuals and communities are disproportionately burdened by cigar smoking and exposed to triggers for smoking these products, more research and resources should be directed toward these communities for cigar product cessation specifically. Neighborhood-based smoking cessation interventions [52] are a viable way to deliver targeted cessation resources to vulnerable communities but have historically focused on cigarette smoking cessation. Additionally, the low harm perceptions and normalization of cigar product use reported by many study participants suggest that culturally appropriate education initiatives are needed to combat these pro-cigar-smoking influences. Future research that investigates the harm and cost of cigar and blunt smoking to individuals’ communities may further help inform the messages and programs aimed at reducing the use of those products in the communities.

Our study results should be considered with the following limitations. First, our data were collected during the COVID-19 pandemic, during which participants might have experienced a higher level of stress than usual [53]. The results related to the perceived community stress and reasons for smoking cigarillos might differ if data were collected pre-pandemic. Second, this study did not directly explore societal-level influences (e.g., racism, gentrification, police brutality) on cigar product and blunt smoking among Black young adults. Given the participant discussions of community stress and the frequent linkage of cigar product and blunt use to stress relief, the potential impact of these stressors is a critical topic for further research. Third, our exploration of blunt use was limited by the omission of questions regarding cannabis acquisition; cannabis availability and acquisition norms may be additional community-level factors with the potential to influence cigar product and blunt smoking behavior.

## 5. Conclusions

Through in-depth interviews, Black young adult cigar product smokers described cigar and blunt smoking as ubiquitous and normative in their communities. Targeted multimodal advertising, socioeconomic strain, easy access to cigar products, and influential cigar product smoking cues perceived in participants’ communities can encourage cigar product and blunt smoking and hinder cessation among Black young adults. Interventions targeting pro-cigar-smoking influences at the community level may be a valuable tool in combatting tobacco-related health disparities.

## Figures and Tables

**Table 1:** Codebook Used for Identifying and Analyzing Themes Emerging from Interviews.

Code *	Definition
Sales and Access	The places and sources from which participants purchased and accessed cigar products; participants’ perceived access to cigar products in their communities
Community Norm and Culture	Perceived community norms and culture related to cigar product smoking and use; reasons for and patterns of cigar and blunt use shared by community members
Marketing Exposure	Information related to store displays and general exposure to marketing of cigar products, including where the cigar marketing materials were placed
Marketing Reaction	Information related to participants’ perceptions of and reactions to the cigar marketing materials and what they recalled seeing from those marketing materials
Smoking Cues	Cues and triggers that promote cigar smoking episodes in participants’ communities

Note *: Within each main code category, subcodes were generated for each cigar product type predominantly smoked by participants and for product differences.

**Table 2:** Participant Characteristics (*n* = 40).

	*n*	%
Age (mean, SD ^1^)	26.0	2.4
Biological Sex		
Male	17	42.5%
Female	23	57.5%
Education Level		
≤GED ^2^ or high school	7	17.5%
Some or completed technical school	9	22.5%
Some college	15	37.5%
≥Bachelor’s degree	9	22.5%
Employment Status		
Full time	19	47.5%
Part time	7	17.5%
Unemployed	11	27.5%
Others	3	7.5%
Financial Situation		
Live comfortably	13	32.5%
Meet needs with a little left	15	37.5%
Just meet basic expenses	12	30.0%
Cigar Product Smoking, Past 30 Days		
Large cigars	24	60.0%
Cigarillos	36	90.0%
Filtered cigars	7	17.5%
Blunts	23	57.5%
Number of Cigar Products Smoked, Past 30 Days		
One product	4	10.0%
Two products	16	40.0%
Three products	11	27.5%
Four products	9	22.5%
Most Frequently Smoked Cigar Product, Past 30 Days		
Large cigars	4	10.0%
Cigarillos	16	40.0%
Filtered cigars	2	5.0%
Blunts	18	45.0%
Use of Other Tobacco Products, Past 30 Days		
Cigarettes	23	57.5%
E-cigarettes	26	65.0%
Hookah	27	67.5%

Note ^1,2^: SD: Standard Deviation. GED: General Educational Development (a high school equivalency diploma in the U.S.).

## Data Availability

Interview transcripts and other relevant data are available upon request.
